# A study on the association of serum fibroblast growth factor-23 with various indices of chronic kidney disease patients not yet on dialysis

**DOI:** 10.15171/jrip.2016.22

**Published:** 2016-04-22

**Authors:** Fatemeh Yaghoubi, Farrokhleghah Ahmadi, Mahboub Lesanpezeshki, Mitra Mahdavi Mazde

**Affiliations:** ^1^Department of Nephrology, Tehran University Medical Sciences, Tehran, Iran

**Keywords:** FGF23, Chronic Kidney Disease, Dyslipidemia

## Abstract

**Introduction:** Fibroblast growth factor-23 (FGF23) is found as a bone derived hormone which can influence the serum levels of parathormone, phosphorous and 1, 25-dihydroxyvitamin D. FGF23 may be a cardiovascular biomarker in patients with chronic kidney disease (CKD). While, FGF23 is inversely correlated with serum adiponectin level, thus it is possible that FGF23 is correlated to fat mass and related to dyslipidemia in CKD.

**Objectives:** In this investigation, we studied the association of serum levels of FGF23 with serum lipid profile, body mass index (BMI), and various cardiovascular risk factors in patients with CKD.

**Patients and Methods:** This is a cross-sectional study, which was conducted on 80 non-dialysis patients (glomerular filtration rate [GFR] <60 cc/min) with CKD. Laboratory results and demographic information recorded in pre-defined data sheets and their relationship with FGF23 were analyzed.

**Results:** Eighty patients were studied. Mean ± standard deviation (SD) of serum creatinine and FGF23 were 2.18 ± 0.73 mg/dl and 15.028 ± 20.20 RU/ml respectively. The correlation between FGF23, low-density lipoprotein cholesterol (LDL-C), high-density lipoprotein cholesterol (HDL-C), ApoA, ApoB, intact parathyroid hormone (iPTH), calcium, phosphorous, alkaline phosphatase, 25-hydroxyvitamin D, BMI, waist girth, serum triglyceride and cholesterol was not significant (*P* > 0.05). In this study, the correlations of FGF23, with serum albumin (*P* = 0.036) and systolic blood pressure (*P* = 0.001) were significant.

**Conclusion:** Our study showed that there are not any significant correlations among FGF23, BMI and lipid profile. There are not any significant correlations between FGF23 and age, sex, and level of calcium, phosphorous, vitamin D. However, the relationships between FGF23 and serum albumin was negatively significant. Additionally we detected a positive significant correlation of FGF23 with level of systolic blood presure.

**Implication for health policy/practice/research/medical education:**

In a study on 80 chronic renal failure patients not yet on dialysis with mean age of 59.82 ± 14.49 years, and serum level of fibroblast growth factor-23 (FGF23) of 15.028 ± 20.20 RU/ml, we found relationships of FGF23 with serum albumin (negative significant correlation) and systolic blood pressure (positive and significant).

## Introduction

The mortality rates in patients with chronic renal failure are higher than the others. Around 50% of these patients die as a result of cardiovascular disease conditions such as pulmonary edema, chronic heart failure, fatal arrhythmias, pericarditis, valvular disease and cardiogenic shock are some of cardiovascular disorders (CVD), as the leading cause of death in these patients. This can be probably due to a higher prevalence of important risk factors such as hypertension, anemia, uremia, disturbances in calcium, phosphorous and sodium, balance, dyslipidemia, atherosclerosis, diabetes mellitus and smoking. Chronic kidney disease (CKD) has been known as a serious condition which has significant complication. CKD is known as a risk factor of disorders such as cardiovascular diseases, electrolyte disturbances, bone disease, cognitive and psychological disorders and many other disease. The nature of this disease is progressive which causes new complications too. On the other hand, there are various parameters which can significantly affect the progress and complications of CKD (1-4).

Terminal stage of chronic renal failure named end-stage renal disease (ESRD). At this stage, patients will need renal replacement therapy. In ESRD patients have lower life expectancy and life quality than general population. Overall mortality rates in these patients are significantly higher than general population. Dialysis and kidney transplantation are two common methods of renal replacement therapy. Availability of the two methods, dialysis quality and support quality for kidney transplantation patients are factors which are strongly related to socio-economic conditions, health care facilities and health care constitutions of the society (5).

## Objectives

In this investigation, we studied the correlation of serum levels of FGF23 with serum lipid profile, BMI and some of the other cardiovascular risk factors in a group of patients with CKD not yet on dialysis.

## Patients and Methods

### Study population

This is a cross-sectional study which was conducted on 80 non-dialysis patients (glomerular filtration rate [GFR] <60 cc/min) with CKD.

### Data collection

Patients’ demographic information and medical history of the study population including age, sex, height, weight, history of smoking, heart disease, hypertension, diabetes, hyperlipidemia and drug history were obtained and recorded in pre-defined data sheets. Blood pressure of the patients were recorded. Laboratory measurements including serum levels of creatinine, triglyceride, total cholesterol, low-density lipoprotein cholesterol (LDL-C), high-density lipoprotein cholesterol (HDL-C), Apo A, Apo B, fasting blood sugar (FBS), serum levels of calcium, phosphorous, magnesium, alkaline phosphatase, intact parathyroid hormone (iPTH), 25-hydroxyvitamin D level, uric acid and albumin were measured after 12 hours fasting before obtaining their blood specimens, using standard kits. Serum FGF23 was assessed by ELISA method (Demeditec, Germany). Waist girth measured and body mass index (BMI) was also calculated by standard method (NIH).

### Exclusion criteria

The exclusion criteria were using steroids, nonsteroidal anti-inflammatory drugs (NSAIDs) or estrogens. Also presence of active or chronic infection and malignancies were the other exclusion criteria.

### Ethical issues

The research followed the tenets of the Declaration of Helsinki. Informed consent was obtained and the research was approved by the Ethics Committee of Tehran University of Medical Science (Shariati hospital).

### Statistical analysis

Quantitative variables were calculated as mean ± standard deviation (SD). *T* test and Mann-Whitney U test was used for analyzing the serum levels of qualitative variables and chi-square and Fisher’s exact test for quantitative variables. *P* value lower than 0.05 was considered statistically significant. The distribution of quantitative data were assessed by one sample Kolmogorov–Smirnov test. Since FGF23 had not a normal distribution, Spearman’s correlation was used for the relation to quantitative variables. Statistical analysis was conducted by SPSS version 18.

## Results

This study included 44 men (55%) and 36 women. The mean age of patients was 59.82±14.49 years.

Cause of CKD among the patients were hypertension 34 (41%), diabetes mellitus 18 (21.7%), glomerulonephritis 10 (12%), renal stones 4 (4.8%), cysts 2 (2.4%) and unknown 15 (18%) (Table 1). The mean, SD, minimum and maximum of all variables and correlation of FGF23’s variables are shown in Tables 2 and 3. Mean ± SD of serum creatinine and FGF23 were 2.18±0.73 mg/dl and 15.028±20.20 RU/ml respectively. 2, 3 and 4 also show the association between FGF23 and other demographic or laboratory parameters. Briefly, correlation of FGF23 with age (*P*=0.55), serum creatinine (*P*=0.19), LDL-C (*P*=0.063), HDL-C (*P*=0.168) and serum triglyceride levels (*P*=0.99) was not significant. Likewise, the association of FGF23 serum with serum cholesterol (*P*=0.63), Apo A (*P*=0.21), and Apo B (*P*=0.74) was not significant. Accordingly, the correlation of FGF23 with intact PTH was not significant (*P*=0.28) (Table 3). The correlation between GFR of all patients (37.59±11.21 cc/min) and serum FGF23 was not significant (*P*=0.11). We categorized GFR into three groups in patients with following prevalence. Group 1 with GFR of 45-59 = 24 cc/min (29.6%), group two with GFR of 30-44 = 34(42%) and group 3 with GFR of 15-29 cc/min = 22 (27.2%). None of the groups had correlation with FGF (*P*>0.05; Table 4).

**Table 1:** Causes of chronic kidney disease

**Causes**	**n (%)**
Hypertension	34 (41)
Diabetes mellitus	18 (21.7)
Glomerulonephritis	10 (12)
Renal stones	4 (4.8)
Cysts	2 (2.4)
Unknown	15 (18)

**Table 2:** Data of the patients

**Variable**	**Mean ± SD**	**Min.**	**Max.**
FGF23 (Ru/ml)	15.028±20.20	0.20	103.40
Age (y)	59.82±14.49	24	83
BMI, kg/m^2^	27.58±5.84	17.6	40.5
Waist girth (cm)	96.84±13.48	62	130
FBS (mg/dl)	105.73±32.68	50	212
GFR (cc/min)	37.59±11.21	6.2	59
Creatinine (mg/dl)	2.18±0.73	1.1	5
LDL (mg/dl)	107.40±37.58	40	230
HDL (mg/dl)	42.56±20.20	18	167
TG (mg/dl)	180.66±100.24	51	751
Cholesterol (mg/dl)	181.16±47.46	71	331
Apo A (g/l)	1.43±0.30	0.6	2.4
Apo B (g/l)	0.90±0.24	0.4	1.7
iPTH (pg/ml)	123.70±145.58	11.50	835
Albumin (g/dl)	4.20±44.00	2.8	5
Calcium (mg/dl)	9.01±0.84	5.4	12
Magnesium (mg/dl)	2.27±0.33	1.1	3
Phosphorous (mg/dl)	3.96±0.93	2.10	7.20
Alkaline phosphatase (IU/l)	246.92±156.66	64	972
Uric acid (mg/dl)	7.46±1.66	4	12.40
BUN (mg/dl)	81.82±39.74	27	281
25-hydroxyvitamin D (ng/ml)	29.95±18.85	3.7	70
SBP (mm Hg)	132.00±15.23	110	190
DBP (mm Hg)	79.94±5.98	65	100

Abbreviations: FGF23, Fibroblast growth factor 23; iPTH, Intact parathyroid hormone; TG, Triglyceride; HDL, high-density lipoprotein; LDL, low-density lipoprotein; FBS, fasting blood sugar; BMI, body mass index; BUN, blood urea nitrogen; SBP, diastolic blood pressure; DBP, diastolic blood pressure. *P*< 0.05 is considered significant.

**Table 3:** Correlation and significant levels between FGF23 and variables

**Variables**	**Correlation rate**	**P**
FGF23 (Ru/ml)		
Age (y)	0.067	0.55
BMI (Kg/m^2^)	-0.036	0.75
Waist girth (cm)	0.15	0.18
FBS (mg/dl)	-0.019	0.87
GFR (cc/min)	0.18	0.11
Creatinine (mg/dl)	0.14	0.19
LDL (mg/dl)	-0.054	0.63
HDL (mg/dl)	-0.156	0.163
Triglyceride (mg/dl)	-0.001	0.99
Cholesterol (mg/dl)	-0.53	0.63
Apo A (g/l)	-0.14	0.21
Apo B (g/l)	-0.038	0.74
IPTH (pg/ml)	-0.12	0.28
Albumin (g/dl)	-0.23	0.036
Calcium (mg/dl)	0.006	0.96
Magnesium (mg/dl)	-0.1	0.37
Phosphorous (mg/dl)	-0.03	0.78
Alkaline phosphatase (IU/L)	0.038	0.74
Uric acid (mg/dl)	-0.064	0.57
BUN (mg/dl)	-0.073	0.51
25-hydroxyvitamin D (ng/ml)	-0.19	0.33
SBP (mm Hg)	0.41	0.001
DBP (mm Hg)	0.18	0.11

Abbreviations: FGF23, Fibroblast growth factor 23; iPTH, Intact parathyroid hormone; TG, Triglyceride; HDL, high-density lipoprotein; LDL, low-density lipoprotein; FBS, fasting blood sugar; BMI, body mass index; BUN, blood urea nitrogen; SBP, diastolic blood pressure; DBP, diastolic blood pressure.

**Table 4:** Mean, SD and correlation of GFR to FGF23

**GFR**	**Min.**	**Max.**	**Mean** ±** SD**	**Pearson’s correlation**	***P***
45-59	0.70	91.90	12.62±17.87	0.11	0.65
30-44	0.20	40.70	11.44±9.90	0.02	0.43
15-29	0.20	103.40	21.72±28.03	0.12	0.21

In this study, the association of FGF23 with systolic blood pressure was positively significant (r=0.41, *P*=0.001, Table 4) and also with serum albumin (r=-0.23, *P*=0.036) was negatively significant.

## Discussion

Vitamin D and PTH have traditionally believed as the main factors in bone and mineral hemostasis, however, recent findings regarding FGF23 and bone-kidney have produced a new point of view in CKD and mineral bone disease. In summary, the most important function of vitamin D and PTH is stabilization of serum calcium levels in a suitable range through induction of 1,25-dihydroxy vitamin D [1,25 (OH)2 D3] synthesis, leading to reabsorption of calcium and reducing urinary excretion of calcium by kidneys. On the other hand, PTH also increase calcium uptake from bones.

PTH induces production of 1, 25 (OH) _2_ D_3_ through increasing CYP27b1, increasing reuptake of calcium in proximal tubules and in distal tubules by regulation of TRPV5. Furthermore, PTH increases the uptake of calcium and phosphate in bones through inducing RANKL by osteoblasts (which in turn stimulate osteoclasts that increase uptake from bones). Additionally, increase of 1,25 (OH)_2_ D_3_ production by kidneys, causes an increase in phosphate and calcium reabsorption by small intestine. Overall, uptake of calcium from bones, decreases in urinary excretion of calcium and increases in its absorption by intestine results to normalizing serum calcium levels. Increasing the uptake of phosphate from bones and by intestines is regulated by PTH and reduces the reuptake of phosphate in renal tubules to balance the levels of phosphate. In fact, FGF23 and bone-kidney axis act as a biological route which have recently been discovered as the main players in CKD-mineral and bone disorder (CKD-MBD). Recent findings showed that osteoblasts and osteocytes are important sites for FGF23 production. FGF23 is excreted by bone, targets kidneys, and regulates phosphate and also vitamin D metabolism. It may also have other functions in regulating parathyroid and phosphate which needs to be studied further (6-8). In this study, we assessed the correlation of serum levels of FGF23 and BMI and lipid profile in patients with CKD. We studied 44 men (55%) and 36 women (45%). The mean age of patients was 59.82±14.49 years and the mean of FGF23 levels was 15.028±20.20 RU/ml. The correlations of FGF23 with BMI, LDL-C, HDL-C, Apo A, Apo B, triglyceride and cholesterol were not significant. Our study also showed that correlations between FGF23 and other factors such as age, sex, calcium, phosphorous and vitamin D were not significant. Our findings are opposed to the study of Mirza et al, which serum levels of triglyceride and total cholesterol positively correlated with serum FGF23. They also found an inverse relationship between Apo A1 and FGF23 (9). The study of Mirza et al showed that the correlation among FGF23 serum levels and BMI and waist girth is stronger than elevation of triglyceride and reduction of HDL-C, Apo A1. They also found that, serum FGF23 were correlated to trunk obesity and total body fat which may reveal a relation between cardiovascular disease and chronic renal failure to the level of FGF23 (9).

Holeck et al showed a correlation between FGF23 and metabolic syndrome in another study. They detected serum value of FGF23 was high in obese and premenopausal women. They concluded that, FGF23 is probably related to the reduction of serum phosphorous in obese patients (10). In animal studies, FGF23 has caused both vascular calcification and glucose and lipid homeostasis disturbances (11). It was also shown that phosphorus reducing therapy with sevelamer in chronic renal failure resulted in lipid improvement including elevation in HDL-C and reduction in LDL-C which can show a relation between bone metabolism and dyslipidemia in CKD. Other families of FGF such as FGF21 have higher levels in obesity, type 2 diabetes and metabolic syndrome (11). In the end-stages of CKD, FGF23 could not reduce serum phosphate and it seems that abnormally high plasma levels of FGF23 cause adverse effects such as LVH, rapid progression of CKD and early mortality (12).

## Conclusion

In our study, only level of blood pressure and serum albumin levels were significantly correlated with FGF23. These require further investigation to better understand of this aspect of CKD patients.

## Limitations of the study

Small proportion of the patients was a limitation of our investigation. Our study was cross-sectional and we could not compare our findings with a control group. Hence, it is suggested to conduct case-control studies with larger sample sizes to better assess the role of FGF23 in renal disease more precisely.

## Acknowledgements

Authors are grateful to research Deputy of Department of Nephrology, Tehran University Medical Sciences, for their financial support of this project.

## Conflicts of interest

The authors report no conflicts of interest. The authors alone are responsible for the content and writing of the article.

## Authors’ contribution

All authors contributed to design of the research. PY, FD and SS conducted the research. SS and AP analyzed the data. PS and AP prepared the manuscript. All authors read, revised, and approved the final manuscript.

## Ethical considerations

Ethical issues (including plagiarism, data fabrication, double publication) have been completely observed by the authors.

## Funding/Support

This paper is extracted from thesis of Dr. Fatemeh Yaghoobi and financial support was provided by the Chronic Renal Failure Research Center of Tehran University of Medical Sciences.
